# What health care services does the public want and who should decide? Ask them!

**DOI:** 10.1186/s13584-016-0107-2

**Published:** 2016-10-10

**Authors:** Adele Diederich

**Affiliations:** Life Sciences & Chemistry, Jacobs University Bremen, Campus Ring 1, D - 28759 Bremen Germany

## Abstract

Most of the parties involved in healthcare decisions – governments, politicians, healthcare professionals, pharmaceutical companies, special interest groups – actively work to make their desires known. In Israel the public is part of the decision committee; in Germany health care decision are made more or less without the public being involved. In a recently published IJHPR article, Giora Kaplan and Orna Baron-Epel raise the question of how well acquainted senior decision makers in the Israeli health system are with the public’s priorities regarding the services being considered for inclusion in the public funding list. This commentary speculates about the reasons for the discrepancies found in that article between the decision makers’ and the public’s view. Furthermore, it reports on survey results from Germany about who should be part of the decision making committee and briefly touches upon the situation in other OECD countries. While public opinion may not be the determining factor, all authors advocate a strengthening of the public’s contribution to the health care decision making process, including steps to make decision makers aware of public priorities on an ongoing basis.

## Background

New expensive health technologies, an aging population and changing epidemiology all increase health care expenditures, and as a result, health systems worldwide are struggling with the need to control costs to maintain system viability. Priority setting in health-care services according to some pre-defined criteria is proposed as one possibility to handle the problem of limited resources. Most of the parties involved in healthcare decisions – governments, politicians, healthcare professionals, pharmaceutical companies, special interest groups – actively work to make their desires known. However, despite their obvious interest in setting priorities, it is the patients who will likely have the greatest difficulty in providing input to these discussions.

If priority-setting decisions are to be accepted, it is important to include the public in the decision-making process [7]. The Ljubljana charter on reforming health care in Europe therefore states that “Health care reforms must address citizens’ needs, taking into account, through the democratic process, their expectations about health and health care” [12]. Legitimizing health policy decisions involves including both the healthy and the sick [1,6].

How can the public be included? Sabik and Lie [9] review priority setting efforts in eight countries (Norway, Sweden, Israel, the Netherlands, Denmark, New Zealand, the United Kingdom, and the state of Oregon in the US). All these countries established specific committees to set priorities in the 1990’s or so and have restructured them since. They strongly advocate public involvement in priority setting in health care but to different degrees. Norway encourages a public discussion; Denmark offers public events and distributed material about priority setting in medicine; New Zealand, the Netherlands, Oregon, and Sweden include feedback from public discussion forums and results from surveys in their decision making process. In England the public can get involved at various levels and a website (National Institute for Health and Clinical Excellence, http://www.nice.org.uk/) offers active participation.

## The situation in Israel

In Israel, the National Advisory Committee decides what should be added to the list of publicly funded health services, with more than one-third of the committee members being public representatives [10]. In this context, Giora Kaplan and Orna Baron-Epel [8] raise the question of how well acquainted senior decision makers in the Israeli health system are with the public’s priorities among the services being considered for inclusion in the funding list. The research was conducted in two steps. First, they conducted a phone population survey regarding the public’s opinion on priorities in health across a variety of services (e.g. fertility treatments, cardiac rehabilitation, nursing care, prevention, mental health). Second, they carried out face-to-face interviews with senior officials in the main institutions of the Israeli health care system with respect to their perceived and expected preferences of the public. The results show a large discrepancy between the public’s preferences and the decision makers’ predicted preferences. Before I will speculate about some reasons for the misjudgments of the decision makers I describe the situation of public involvement in Germany.

## The situation in Germany

In Germany discussions of priority setting – with or without the public being involved – are carried out mainly in academia. Several attempts have been made to include decision makers – particularly physicians and politicians - without success. Indeed, all ministers of health over the last decades or so have refused to even talk about this issue. For those insured under statutory health insurance (SHI), which is about 90 % of the population, the Federal Joint Committee (Gemeinsamer Bundesausschuss, G-BA) makes decisions on healthcare benefits, and it defines in detail what adequate, appropriate, and economical healthcare, as defined by law, entails (https://www.g-ba.de/institution/service/publikationen/gba/).

The Federal Joint Committee (G-BA) consists of five stakeholder groups with voting rights (three impartial members; five representatives from the Central Federal Association of Health Insurance Funds, the organization representing all statutory health insurance funds; five representatives from the Central Federal Association of Health Insurance Funds, the organization representing all statutory health insurance funds; two representatives from the National Association of Statutory Health Insurance Physicians, which includes all licensed physicians and psychotherapists who treat SHI patients; two representatives from the German Hospital Federation, the interest group representing hospitals; and one representative from the National Association of Statutory Health Insurance Dentists, which includes all licensed dentists who treat SHI patients). Five patient representatives take part in all plenary sessions, subcommittee meetings, and workgroup meetings. They are entitled to submit petitions and take part in discussions, but have no right to vote. This would be the main source of information on the (sick) public’s preferences. Note, the G-BA decides which services are brought to the committee and decides what is eventually funded.

**Fig. 1:**
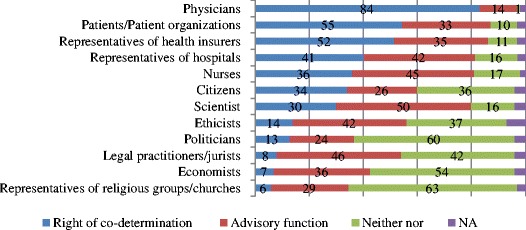
Citizens’ preferred stakeholders’ involvement in medical decision processes. The public’s agreement to the question: “In your opinion who should take part in decision making when deciding what should be paid by the SHI; who should only have an advisory function; and who should not have a say at all?” The results are in percentage. NA stand for “no answer given”

How does the public feel about this? A population survey (computer assisted personal interviews (CAPI), *n* = 2031) was conducted in 2009 addressing thirty-four questions with 135 items organized into ten health care and health system related themes (e.g., [4,5]). One theme was about the decision makers and their function in distributing health care benefits. The question and results (percentage of agreement) are shown in the following Fig. 1.

According to public opinion, representatives of health insurers (52 %), physicians (84 %) und patients/patient organization (55 %) should have a right of co-determination, when deciding what health care services should be paid for by the statuary health insurance. At the same time, a high percentage of the public indicate that the following should have *no* say: politicians (60 %), representatives of religious groups/churches (63 %) and economists (54 %). This is interesting in so far as these stakeholder groups do have considerable influence when it comes to health care services, directly (such as political programs, e.g. personal responsibility –out of pocket payment) or indirectly (like ethics committee, e.g. prenatal diagnosis). Respondents expressed the view that some stakeholders (representatives from hospitals, nurses, scientists) should rather be co-determining and advisory, whereas other stakeholders (ethicists and jurists) should have at most advisory functions. The respondents are divided when it concerns their own involvement in the decision process. The percentages in all three categories (right of co-determination; advisory function; neither nor) are similar. For no other group can we observe a dichotomy of “right of co-determination” versus “no function”. In an “Others” response category (not listed here) the “general public” and “member of the family” were mentioned. This suggests that some of the respondents assumed only selected citizens for the category “citizens” and not the general public. Obviously, also here we can observe a discrepancy between the public’s opinion and the policies employed.

## Possible reasons for decision makers’ misperceptions of public preferences and possible remedies

Kaplan and Baron-Epel contemplate several possible reasons for the lack of accuracy in the decision makers’ predictions on public’s preferences, which include ignoring the input of the public and misinterpreting information presented in the media. The latter could be due to a cognitive bias [11]: If the media report on one particular issue very often, e.g., hospitalization in a private hospital, the decision maker might get the impression that this is highly rated in the public’s opinion. Furthermore, it is possible that the decision makers exhibit a self-serving bias; they may be projecting their own preferences onto the public’s view.

How can we involve the public better, elicit reliable opinions, and increase decision maker awareness of public preferences? For the general public, representative surveys are a means – basically the only means. The argument that they are invalid would also be true for general elections. Over the last decade or so, several representative surveys have been conducted in several countries. The results are mostly discussed in academic communities only; decision makers should be willing to consult them, and their exposure to public survey results should be a part of the decision making process. Town hall meetings and focus groups are applicably only for specific topics with very few participants. They might provide additional information but always face the problem of legitimacy (if not elected). Web based discussion forums like NICE in the UK are another source of information about the public’s preferences regarding health care services.

## The opposition to public involvement

But there are also opponents to public involvement. Bruni et al. [1] list frequently cited reasons for why citizens are (and should be) excluded from the decision process, one being the lack of objectivity and self-serving biases. However, there is no reason to assume that this is different from other stakeholder groups involved in setting priorities in health care, such as physicians, hospital representatives, or representatives from pharmaceutical companies. And the study by Kaplan and Baron-Epel supports this. For instance, citizens prioritized cardio-rehabilitation, which deems to be very reasonable when it comes to surviving or not, whereas the decision makers prioritized fertility treatment, which might reflect a particular ethical/religious attitude. Another argument is the belief that the public’s lack of knowledge about scientific, clinical, and administrative aspects of health care means they cannot contribute meaningfully to priority setting. However, as Bruni et al. [1] point out, the public has real-life experience as users of the health care system and can offer insight into the values and beliefs of the public at large.

In agreement with Kaplan and Baron-Epel, I strongly support public involvement when it comes to setting priorities in health care. After all, it is the public which finances the health system (by premiums and taxes) and primarily uses the services; they are the largest and most important stakeholder group. The Israeli process in which public involvement is guaranteed by the composition of the Advisory Committee - even if the list of preferred benefits needs to be debated - seems to merit acceptance from the Israeli public and physicians [2,3]. At the same time, we cannot assume that the “public representatives” appointed by Israel’s Minister of Health will responsibly and accurately give voice to the priorities of the general public; much depends on how they are selected and whether, as Kaplan and Baron-Epel suggest, they are provided with periodic and systematic data about public preferences.

## Conclusions

The comparison between Germany and Israel suggests that citizen views on priority setting should be addressed by policy makers in two layers. The first layer is to recognize that citizens have preferences and desire citizen input into decision making processes. The second is to ensure that decision makers are aware of public preferences and public views on important decisions. This requires involving public representatives in the decision making process, ongoing gathering of data from the public, passing the data onto decision makers, and re-checking citizen views on priority setting decisions and the process by which these are made.

## Abbreviations

G-BA: Gemeinsamer Bundesausschuss (Federal Joint Committee)
NICE: National Institute for Health and Clinical Excellence
SHI: Statutory health insurance
WHO: World Health Organization
